# High prevalence of diagnosis of diabetes, depression, anxiety, hypertension, asthma and COPD in the total population of Stockholm, Sweden – a challenge for public health

**DOI:** 10.1186/1471-2458-13-670

**Published:** 2013-07-18

**Authors:** Axel C Carlsson, Per Wändell, Urban Ösby, Ramin Zarrinkoub, Björn Wettermark, Gunnar Ljunggren

**Affiliations:** 1 Centre for Family Medicine, Department of Neurobiology, Care Sciences and Society, Karolinska Institutet, Alfred Nobels Allé 12, 141 83 Huddinge, Sweden; 2 Department of Public Health and Caring Sciences/ Section of Geriatrics, Uppsala University, Uppsala, Sweden; 3 Neurogenetics Unit, Department of Molecular Medicine and Surgery, Karolinska Institutet, Solna, Sweden; 4 Department of Psychiatry, Tiohundra AB, Norrtälje, Sweden; 5 Public Healthcare Services Committee Administration, Stockholm County Council, Box 6909, SE- 102 39 Stockholm, Sweden; 6 Centre for Pharmacoepidemiology and Department of Laboratory Medicine, Division of Clinical Pharmacology, Karolinska University Hospital, Huddinge, Sweden; 7 Medical Management Centre, Department of Learning, Informatics, Management and Ethics, Karolinska Institutet, Berzelius väg 3, SE-17177 Stockholm, Sweden

**Keywords:** Administrative databases, Primary care, Gender differences, Age differences, Epidemiology

## Abstract

**Background:**

There is limited knowledge on the prevalence of disease in total populations. Such studies have historically been difficult to conduct but the development of health data registers has facilitated large-scale studies on recorded diagnoses in entire regions. The aim of this study was to analyze the prevalence of diagnosis of six common diseases in the Swedish capital region.

**Methods:**

The study population included all living persons who resided in Stockholm County, Sweden, on December 31^st^ 2011 (N = 2 093 717). Information on all consultations between 2007 and 2011 was obtained from primary health care, specialist outpatient care and inpatient care. Prevalence was defined as the proportion of individuals with a recorded diagnosis of diabetes, depression, anxiety disorders, hypertension, asthma and chronic obstructive pulmonary disease during the five year period, respectively. Analyses were done by age and gender.

**Results:**

Hypertension had the highest five-year prevalence (12.2%), followed by depression (6.6%), diabetes mellitus (6.2%), asthma (5.9%), anxiety disorders/phobia (4.8%), and COPD (1.8%). Diabetes was more common in men (5.3% of women and 7.1% of men) while depression (8.7% in women and 4.4% in men) and anxiety (6.3% in women and 3.4% in men) were considerably more common in women. Smaller gender differences were also found for hypertension (13.0% in women and 11.4% in men), asthma (6.4% in women and 5.4% in men) and COPD (2.1% in women and 1.6% in men). Diabetes, hypertension and COPD increased markedly with age, whereas anxiety, depression and asthma were fairly constant in individuals above 18 years. During one year of observation, more than half of all patients had only been diagnosed in primary health care, with hypertension being the diagnosis with the largest proportion of patients only identified in primary health care (70.6%).

**Conclusion:**

The prevalence of common diseases in the population can be estimated by combining data gathered during consecutive years from primary care, specialist outpatient care and inpatient care. However, accuracy of disease prevalence is highly dependent on the quality of the data. The high prevalence of the six diagnoses analysed in this study calls for preventive action to minimize suffering and costs to society.

## Background

Epidemiological data on the prevalence and incidence of diseases are valuable for making policy decisions and promoting evidence-based disease prevention and management. Prevalence can be assessed in several ways [1]. First, by self-reported presence of diseases, which may contain several uncertainties and result in different validity depending on the studied diagnosis. Second, by using or combining healthcare source data based on diagnosis records or on drug prescriptions but where individuals without prescribed drugs will be lost. Third, by the use of two or more data sources which could be combined by capture–recapture methods. Fourth, by population-based screenings which will identify new cases but will have lower validity in studies with low participation rates.

The establishment of the Swedish Hospital Discharge Register in 1964 has facilitated studies on the prevalence of diseases that require hospitalization [2,3]. The Swedish Hospital Discharge Register includes all hospitalizations from 1987 onwards as well as all outpatient consultations in hospitals from 2001 onwards. A number of studies using this register have showed that the quality of its data is high and the register has been widely used in many outcome studies [4,5].

In recent years, the development of electronic medical records and administrative databases has facilitated studies on the prevalence and incidence of diseases in the total population of different regions [6,7]. The main advantages of these registers are their full coverage of healthcare consultations and that they are relatively easy and inexpensive to use. Disadvantages include possible bias in recording of diagnoses and variability in data quality [8].

In Stockholm County, with a total population of more than two million people, all diagnosis codes and reasons for hospitalizations and consultations in primary health care and specialist care are recorded and stored in a large administrative database. This comprehensive data collection enables epidemiological research in a large unselected population cohort. The primary aim of this study was to use these data to estimate the prevalence of six common diagnoses, namely diabetes mellitus, depression, anxiety disorders, hypertension, asthma, and chronic obstructive pulmonary disease (COPD) in the total population of Stockholm County. Furthermore, the distribution of these diagnoses across the different sectors of the healthcare system, i.e. primary health care (PC), specialist outpatient care (SOC) and inpatient care (IC), recorded during the last year of observation, 2011, was also determined.

## Methods

Stockholm County has 2.1 million inhabitants, representing more than one-fifth of Sweden’s entire population. This area of Sweden includes the capital city of Stockholm and several other cities and towns, as well as large rural areas and a sparsely-populated archipelago. The Stockholm County Council is responsible for financing primary and secondary health care, mainly through taxes. Besides illegal immigrants, the general health insurance covers all residents. The majority of services are provided by County-owned facilities. However, during the last decade, more than 50% of primary care and approximately 8% of acute hospital care services have been outsourced to private providers, either through tender processes or managed patient choice [9]. Private providers are in contractual agreements with the County and are legally obliged to record diagnoses and file reports to the authorities just like public providers [10]. With the exception of very few private clinics that operate without subsidies in Stockholm, all consultations and diagnoses are recorded and stored in the so-called VAL, a central database. Besides consultations and diagnoses, VAL compiles and stores data on healthcare utilization and socio-demographics. The database has been used for healthcare planning, practice remuneration and quality assessment since the beginning of the 1980s, and its content, registration routines, and supporting software have improved over the years. It has previously been used as a source of information in a number of scientific studies, e.g. studies of hip fractures and its co-morbidities [11,12], and Parkinson’s disease [13]. As an indication for its accuracy and validity, VAL is used by the Council for updating the National Patient Register kept by the Swedish National Board of Health and Welfare (NBHW) as well as the annual benchmarking reports of the NBWH and the Swedish Association of Local Authorities and Regions [4].

VAL has more than 99% coverage of hospital care. More specifically, for each hospital stay the VAL database contains a record of the provider unit, an encrypted patient identification number, age and sex, the type and length of the stay, up to ten diagnoses given, and ten interventions (primarily surgical procedures, transfusions, anaesthetic procedures). Since 1997, diagnoses have been coded according to WHO’s International Classification of Diseases, 10^th^ edition (ICD-10) and procedures classified according to the Nordic Classification of Surgical Procedures (NCSP).

Reporting utilization in specialized ambulatory care, whether in hospital clinics or other locations, is also mandatory since the late 1990s. This includes reporting diagnoses (up to eight) for consultations by a physician, date, type of visit, and certain procedures performed. Also, nurse visits in homecare, visits to occupational or physical therapists as well as most other healthcare professionals are registered. VAL has more than 90% coverage of utilization in specialized ambulatory care.

Storing data on primary care diagnoses in VAL has a shorter history. In 2003, a project to extract information on diagnoses (up to 15) from the electronic medical records, when available, was launched. It has been estimated that approximately 85% of all diagnoses in primary care are stored in VAL.

All data extracted from VAL were anonymised in the current study.

### Ethics

All data we handled were anonymized and none of the numbers presented could be traced back to any individual. Management and analysis based on the VAL database is a part of continuous quality control of healthcare utilization in Stockholm county council. Research projects deemed as quality control are not subject to ethical research board decisions in Sweden.

### Study population

The present study population included all living persons who resided in Stockholm County on December 31^st^ 2011 (N = 2 093 717). Demography of Stockholm County and number of doctor visits per person in 2011 are shown in Table 1. Residents who died or moved from the region during the five-year period of interest (2007–2011) were excluded. Data on all healthcare consultations in PC, SOC and IC between 2007 and 2011 were extracted from VAL.

**Table 1:** The demography and number of doctor visits in Stockholm county as of December 31, 2011

**Age-groups**	**Women 2011**		**Doctor visits per woman**		**Men 2011**		**Doctor visits per man**		**Total 2011**		**Doctor visits per person**	
	**n**	**%**	**PC**	**SOC**	**n**	**%**	**PC**	**SOC**	**n**	**%**	**PC**	**SOC**
0-17	219612	20.8	1.62	0.77	231845	22.4	1.57	0.89	451457	21.6	1.59	0.83
18-44	403774	38.2	1.69	1.24	410537	39.6	0.94	0.73	814311	38.9	1.31	0.98
45-64	254622	24.1	2.13	1.49	255355	24.6	1.57	1.26	509977	24.4	1.85	1.38
65-74	94586	9.0	3.11	2.06	87093	8.4	3.02	2.22	181679	8.7	3.07	2.13
75-84	52699	5.0	5.20	2.64	37948	3.7	5.26	3.01	90647	4.3	5.23	2.79
85-	31383	3.0	6.09	2.23	14263	1.4	6.46	2.89	45646	2.2	6.20	2.44
Total	1056676	100	2.22	1.37	1037041	100	1.64	1.14	2093717	100	1.93	1.26

*PC* primary care, *SOC* specialist open care.

### Major diagnosis groups

The following ICD codes were used to define each major diagnosis group: diabetes mellitus E10-E14, depression F32-F33, anxiety disorders F40-F41, hypertension I10-I15, asthma J45-J46, and chronic pulmonary respiratory disease (COPD) J41-J44, J47. The five-year prevalence of each diagnosis was calculated by dividing the number of persons with any of the diagnosis groups recorded at least once between January 1^st^ 2007 and December 31^st^ 2011 with the total population number as for December 31st 2011. In a similar fashion, one-year prevalence of each diagnosis (2011) was calculated by dividing the number of persons with any of the diagnosis groups recorded at least once during 2011 with the total population number. The one-year- and five-year prevalence figures may be viewed as a period prevalence, especially for depression, and anxiety disorders.

### Statistical methods

Standard descriptive statistics such as numbers and percentages out of the total population (N) were used. Prevalence was presented as total/overall and was also stratified by age and sex. Statistical analysis and data management was performed using SAS software, version 9.3 (SAS Institute Inc., Cary, NC).

## Results

### Demography of Stockholm county

The population of Stockholm county was younger than in the rest of the country, with only 14.2% of the population being 65 years of age and older, and as many as 38.9% in the age group 18–44 years (Table 1).

### Prevalence of diagnosed diabetes mellitus

In total, 5.3% of women and 7.1% of men in Stockholm were diagnosed with diabetes during the five-year period as shown in Table 2A. The highest prevalence was found among persons aged 75–84 years, 23.1% among women and 31.9% among men. In total, 56% of all patients identified with a diabetes diagnosis during a five-year period were diagnosed during the last year of observation, 2011.

**Table 2:** The prevalence and the percentage in the population (Dec. 2011) of six diagnosis groups in 2007–2011 in men, women and all inhabitants (total) in Stockholm county by age: A) diabetes mellitus (E10-E14), B) depression (F32-F33), C) anxiety disorders/phobia (F40-F41) D) hypertension (I10-I15), E) asthma and F) (J45-J46) chronic obstructive pulmonary disease (COPD) (J41-J44, J47)

**Age-groups**	**Women 2007-2011**		**Men 2007-2011**		**Total 2007-2011**		**Recorded diagnosis in 2011 alone and in relation to those recorded between 2007-2011**	
	**N**	**%**	**n**	**%**	**n**	**%**	**n**	**%**
A) The prevalence of diagnosed diabetes mellitus.								
0-17	676	0.3	852	0.4	1528	0.3	1229	80.4
18-44	4918	1.2	6221	1.5	11139	1.4	6700	60.1
45-64	16997	6.7	28212	11.0	45209	8.9	25290	55.9
65-74	14751	15.6	22230	25.5	36981	20.4	20828	56.3
75-84	12179	23.1	12123	31.9	24302	26.8	13228	54.4
85-	6417	20.4	3665	25.7	10082	22.1	4915	48.8
Total	55938	5.3	73303	7.1	129241	6.2	72190	55.9
B) The prevalence of diagnosed depression.								
0-17	570	0.3	265	0.1	835	0.2	640	76.6
18-44	40762	10.1	20131	4.9	60893	7.5	23834	39.1
45-64	32315	12.7	17403	6.8	49718	9.6	17724	35.6
65-74	8470	9.0	4080	4.7	12550	6.9	4313	34.4
75-84	5848	11.1	2344	6.2	8192	9.0	2993	36.5
85-	3817	12.2	1121	7.9	4938	10.8	1592	32.2
Total	91782	8.7	45344	4.4	137126	6.6	51096	37.3
C) The prevalence of diagnosed anxiety disorders/phobia.								
0-17	1035	0.5	639	0.3	1674	0.4	925	55.3
18-44	34551	8.6	18816	4.6	53367	6.6	21919	41.1
45-64	20148	7.9	11421	4.5	31569	6.2	11758	37.2
65-74	5265	5.6	2375	2.7	7640	4.2	2808	36.8
75-84	3380	6.4	1083	2.9	4463	4.9	1704	38.2
85-	2203	7.0	474	3.3	2677	5.9	982	36.7
Total	66582	6.3	34808	3.4	101390	4.8	40096	39.5
D) The prevalence of diagnosed hypertension.								
0-17	115	0.1	121	0.1	236	0.1	71	30.1
18-44	5318	1.3	6047	1.5	11364	1.4	5715	50.3
45-64	41933	16.5	44822	17.5	86755	17.0	49645	57.2
65-74	38685	40.9	37568	43.1	76253	42.0	46191	60.6
75-84	31269	59.3	21367	56.3	52636	58.1	31832	60.5
85-	20498	65.3	8467	59.4	28965	63.5	16052	55.4
Total	137817	13.0	118392	11.4	256209	12.2	149506	58.4
E) The prevalence of diagnosed asthma.								
0-17	17293	7.9	26184	11.3	43477	9.6	17924	41.2
18-44	21646	5.4	14950	3.6	36596	4.5	11945	32.6
45-64	15888	6.2	9167	3.6	25055	4.9	9142	36.5
65-74	6523	6.9	3327	3.8	9850	5.4	3793	38.5
75-84	3978	7.6	1691	4.5	5669	6.3	2217	39.1
85-	1945	6.2	637	4.5	2582	5.7	1023	39.6
Total	67273	6.4	55956	5.4	123229	5.9	46044	37.4
E) The prevalence of diagnosed COPD.								
0-17	335	0.2	434	0.2	769	0.2	455	59.2
18-44	1001	0.3	748	0.2	1749	0.2	721	41.2
45-64	6154	2.4	4685	1.8	10839	2.1	4910	45.3
65-74	6875	7.3	5226	6.0	12101	6.7	6190	51.2
75-84	5388	10.2	3741	9.9	9129	10.1	5027	55.1
85-	2569	8.2	1435	10.1	4004	8.8	2094	52.3
Total	22322	2.1	16269	1.6	38591	1.8	19397	50.3

The individuals with a reported diagnosis in 2011 are also shown in total and as percentage of the individuals with these diagnoses during the full period 2007–2011.

### Prevalence of diagnosed depression

With the exception of children and adolescents (aged 0–17 years) where few were diagnosed (0.18%), a diagnosis of depression was common across all age groups in both men and women as shown in Table 2B. The highest five-year period prevalence of diagnosed depression was found among women aged between 45–64 years (12.7%) and men aged 85 years and older (10.8%). In total, 37% of all patients identified with a depression diagnosis during a five-year period were diagnosed during the last year of observation, 2011.

### Prevalence of diagnosed anxiety disorders / phobia

More women than men were diagnosed with anxiety (6.3% vs. 3.4%) as shown in Table 2C. The highest prevalence was found among individuals aged between 18–44 years. When stratified by sex, 8.6% of women and 4.6% of men were diagnosed of anxiety/phobia recorded. In total, 40% of all patients identified with an anxiety/phobia diagnosis during a five-year period were diagnosed during the last year of observation, 2011.

### Prevalence of diagnosed hypertension

As shown in Table 2D, 13.0% of women and 11.4% of men in Stockholm a hypertension diagnosis was recorded. An increase in diagnosed hypertension with advancing age, ranging between 0.05% in children and 63.5% in those aged 85 years and older was seen. Overall, hypertension was more common in women than men. In total, 58% of all patients identified with a hypertension diagnosis during a five-year period were diagnosed during the last year of observation, 2011.

### Prevalence of diagnosed asthma

As shown in Table 2E, diagnosed asthma was equally common in men and women (6.4% vs 5.4%) and most prevalent in children (9.6%). In total, 37% of all patients identified with a asthma diagnosis during a five-year period were diagnosed during the last year of observation, 2011.

### Prevalence of diagnosed COPD

Diagnosed COPD was more prevalent among persons aged 45 years and older with the highest prevalence in men and women aged between 75–84 years (10.1%) as shown in Table 2F. In total, 50% of all patients identified with a COPD diagnosis during a five-year period were diagnosed during the last year of observation, 2011.

### Distribution of diagnoses recorded across different sectors of the healthcare system

The proportion of patients diagnosed with diabetes, depression, anxiety disorders, hypertension, asthma and COPD in PC, SOC, and/or IC during 2011 varied depending on the diagnosis group. A majority of the patients in all six diagnosis groups were only identified in only PC, with depression (56.5%) and hypertension (70.6%) being the most commonly recorded diagnoses in PC followed by diabetes mellitus (56.1%), asthma (55.9%), COPD (53.6%), and anxiety/phobia (51.6%). When comparisons in the distribution of all six diagnosis groups only in SOC were made, COPD was the most commonly recorded diagnosis (33.8%). In IC, COPD was the most common diagnosis recorded of the six (15%). It was uncommon to have a diagnosis recorded in all three health care sectors. Figures showing the distribution of diagnoses recorded in PC, SOC and IC can be viewed, Additional file 1: Figure S1.

## Discussion

The current study estimates prevalence of diagnosed common diseases in the total population in Stockholm County, a region with more than 2 million inhabitants, using recorded diagnoses in regional registers. The high prevalence of the six common diagnosis groups reported in this study calls for preventive action to be taken. Still, there may be people who are not yet diagnosed and population based health assessments have yielded individuals with newly diagnosed hypertension and diabetes [1,14]. With more than 30% of men and 20% of women aged between 75–84 years being diagnosed with diabetes during a five-year period, considerable amounts of health care resources will be needed. In addition, with 55% of all men and women in this age-group being diagnosed with hypertension, a considerable burden could be expected as this group would likely develop cardiovascular complications. The large amounts of younger patients diagnosed with these conditions, subsequently developing complications, may also pose a challenge for the future.

The fact that the population is large enables stratification by sex and age. Bearing in mind that approximately 20% of the inhabitants of Stockholm County are immigrants [15], the prevalence of common diseases in an urban population of a Western country can be used as a proxy for the prevalence of these diagnosed diseases in similar populations of other regions or countries.

The overall prevalence of diagnosed COPD was 1.8%, which was higher than a previous study of COPD prevalence in a population living in a rural region of Sweden (1.2%) that had a similar design [7]. The difference in prevalence could be due to more particles in the air in Stockholm, higher proportion of smokers or a higher awareness of COPD in recent years. Prevalence of diagnosed COPD increased dramatically among persons aged between 45–84 years whereas it decreased among persons aged 85 years and over which may be explained by the effect of smoking on longevity [16]. The opposite was observed for the prevalence of anxiety /phobia which was high in young adults aged between 18–44 years (6.6%). A recent Swedish study, where a population based sample of 75-year-old persons was interviewed over a period of one month, reported that 3.7% had generalized anxiety disorder according to ICD-10 [17], which is consistent with the five-year prevalence of 4.2% among persons aged between 65–74 years of the total population of Stockholm County reported in this study. Another Swedish study reported that as much as 24% of the Swedish population aged 20–64 years fulfilled the criteria for anxiety and/or depression [18].

Diabetes is often regarded as equally common among men and women, although a male preponderance has been observed in those aged 45 years and over [19]. Wirehn et al. (2007) found an overall male preponderance in diagnosed diabetes prevalence (4.6% in men vs 4.1% in women) [7] while in the current study an even larger male predominance in diagnosed diabetes prevalence was found (7.1% in men vs 5.3% in women). In a review (2001), Gale and Gillespie concluded that men may be more susceptible to physical inactivity and obesity than are women [20]. Overall prevalence of diagnosed diabetes was higher in the current study (6.2%) than in earlier studies in Sweden (4.5%) [7,21]. One explanation for the discrepancy may be the higher rate of immigrants living in Stockholm County [22]. A population-based study has shown that two thirds of the 60-year old diabetes patients are known [15].

The five-year period prevalence of diagnosed depression was high among adults, ranging between 9.0% and 12.2% in women and between 4.7 and 7.9% in men. The higher prevalence of depression among women than among men has been reported previously [23], and may be due to that women may be more active in seeking health care when they experience depressive symptoms. Also, the prevalence of diagnosed depression was lower among individuals younger than 65 years of age than those aged between 65–74 years which may be explained by lack of incentive to be sick-listed in the years following retirement. Persons with depression have been shown to have a higher risk for somatic diseases than non-depressed individuals [24], which may have had an effect on the diagnosed prevalence of all diagnosis groups in women in this study.

Studies of hypertension in the US have shown that the prevalence in the adult population (18 years of age and over) was nearly 30% [25,26], which was higher than that we reported in this study (12.2%) implying there may be a large number of undiagnosed individuals in the total population of Stockholm County. This is in agreement with a screening study of 60 year-old persons from Stockholm which showed that newly diagnosed hypertension was more prevalent than already diagnosed hypertension [14]. The prevalence of hypertension is highly dependent on age [27], which was consistent with our findings. It has been previously shown that prevalence of hypertension is lower in women than in men until menopause, after which prevalence increases and reach levels observed in men [28,29]. Large scale population-based investigations are, however, still sparse and the true prevalence in the population is therefore not fully known, making the findings of the current valuable. Moreover, hypertension awareness, treatment and control vary greatly in different studies from around the world [30], and in populations of different countries studied following the same methodology [31].

In contrast to the other diagnosis groups, asthma appeared to affect children the most. Furthermore, diagnosed asthma was more common in boys than in girls and in women than men. The prevalence rates and gender differences are consistent with a recent review estimating the overall prevalence of asthma at 5-7% in the total population and 8-10% in children [32]. Previous studies have also shown that women with asthma seek care more often than do men [33,34]. Asthma in children is often associated with rhinitis and eczema as well. The BAMSE birth cohort reported that 58% of 12 year-old children had one, two or all these three conditions at some time [35].

Our study revealed important information on the prevalence of diagnosed common diseases in the population. It could be useful for the planning of healthcare needs, resource allocation and disease prevention. Access to electronic longitudinal data from primary healthcare may also provide unique opportunities for performing post-marketing comparative effectiveness research [2]. Some benefits include availability of large populations at a relatively low cost and shortening the time necessary to identify a sufficient number of patients with a specific diagnosed disease. They may also enable studies of patient groups that are usually omitted from randomized controlled trials, i.e. patients with co-morbidities [36]. Recently, prescription data on individuals, using the same encrypted identification numbers as data from VAL, have been added to the database. This enables a unique possibility to link drug prescriptions to diagnoses and patient outcome.

It is important to emphasize that the frequency and diagnosis coverage may vary between different physicians, primary health care centres and diagnosis group [37]. Additionally, the reporting of diagnoses may change along with changes in the healthcare reimbursement system, however, no major changes occurred in the region during the course of this study. To achieve better prevalence estimates, data from PC, SOC and IC could be used together and from consecutive years [7]. The applicability of our method may also vary between different settings. Health care systems vary widely across different regions and countries. More patients in Stockholm receive their care from specialists other than GPs, due to an proportionally smaller primary care sector compared with the UK [38].

Inclusion of only those who were alive at the end of the study period has likely introduced a survival bias as individuals who died during the five-year period were likely to have used considerably more healthcare resources than average. Consequently, the prevalence of diseases that are common among older persons, such as COPD, may have been underestimated. However, this is not a problem if the data are interpreted as point prevalences.

Most of the conditions encountered in PC are readily treatable, for example acute infections and inflammatory conditions, whereas others are chronic or relapsing and often non-acute such as diabetes mellitus, depression, anxiety disorders, hypertension, asthma, and chronic obstructive pulmonary disease (COPD). The diagnostic accuracy may also vary, depending on the disorder and the diagnostic criteria applied. For diabetes, highly valid diagnostic criteria make the disease easy to identify [39]. In the current study we did not make a distinction between Type I and Type II diabetes, as one of the most commonly used diagnosis codes in primary care is diabetes mellitus not otherwise specified. However, it is well known than Type II diabetes is more prevalent than Type I diabetes. While data on asthma, hypertension and diabetes diagnoses the diagnostic accuracy could be expected to be of high validity, data on COPD, anxiety/phobia, and depression may not be as straightforward.

Stockholm County has seen more people moving in than moving out. Consequently, a slight underestimation in prevalence of the diagnostic groups may be expected as new inhabitants may have not yet lived in Stockholm long enough to have diagnoses recorded during the five-year period of interest. This may hold true for individuals aged between 18–44 years. However, this effect may have been balanced out by excluding those who died during the same period.

## Conclusion

In conclusion, the prevalence of known and diagnosed diseases can be estimated using administrative health data registers. Whether a diagnosis is evaluated and recorded in primary care, specialist outpatient care or inpatient care, the diagnosed prevalence estimates are highly dependent on the quality of the diagnosis. It takes further validation studies to investigate if there are great discrepancies in diagnosis accuracy between different levels of care, and how that influences our overall knowledge of the existence of various patient groups. The high prevalence of the six diagnoses analysed in this study calls for preventive action to be taken to minimize suffering and costs to society.

## Competing interests

The authors of this manuscript have no conflict of interest to disclose.

## Authors’ contributions

GL researched data, edited manuscript, contributed to discussion, ACC wrote manuscript, researched data, contributed to discussion. PW, BW, RZ, and UÖ edited manuscript, contributed to discussion. All authors read and approved the final manuscript.

## Pre-publication history

The pre-publication history for this paper can be accessed here:

http://www.biomedcentral.com/1471-2458/13/670/prepub

## Supplementary Material

Additional file 1: Figure S1 Wenn diagrams of reported diagnoses: A) diabetes, C) depression, D) anxiety/phobia, E) hypertension F) asthma, and G) COPD. Primary care = PC, Specialist outpatient care = SOC, Inpatient care = IC. Click here for file

## References

[B1] Wandell PE Johansson SE Gafvels C Hellenius ML de Faire U Sundquist J Estimation of diabetes prevalence among immigrants from the Middle East in Sweden by using three different data sources Diabetes Metab 2008 34 4 Pt 1 328 333 18539497 10.1016/j.diabet.2008.01.012

[B2] Rodriguez EM Staffa JA Graham DJ The role of databases in drug postmarketing surveillance Pharmacoepidemiol Drug Saf 2001 10 5 407 410 10.1002/pds.615 11802586

[B3] Sweden S Statistical yearbook of Sweden 2011 2010 Stockholm: Statistics Sweden

[B4] Ludvigsson JF Andersson E Ekbom A Feychting M Kim JL Reuterwall C Heurgren M Olausson PO External review and validation of the Swedish national inpatient register BMC Publ Health 2011 11 450 10.1186/1471-2458-11-450 PMC3142234 21658213

[B5] Rosen M National health data registers: a Nordic heritage to public health Scand J Public Health 2002 30 2 81 85 12028856 10.1080/140349401753683444

[B6] Nilsson G Ahlfeldt H Strender LE Textual content, health problems and diagnostic codes in electronic patient records in general practice Scand J Prim Health Care 2003 21 1 33 36 10.1080/02813430310000537 12718458

[B7] Wirehn AB Karlsson HM Carstensen JM Estimating disease prevalence using a population-based administrative healthcare database Scand J Public Health 2007 35 4 424 431 10.1080/14034940701195230 17786807

[B8] Sorensen HT Sabroe S Olsen J A framework for evaluation of secondary data sources for epidemiological research Int J Epidemiol 1996 25 2 435 442 10.1093/ije/25.2.435 9119571

[B9] Ekman I Wolf A Olsson LE Taft C Dudas K Schaufelberger M Swedberg K Effects of person-centred care in patients with chronic heart failure: the PCC-HF study Eur Heart J 2012 33 9 1112 1119 10.1093/eurheartj/ehr306 21926072 PMC3751966

[B10] Burstrom B Will Swedish healthcare reforms affect equity? BMJ 2009 339 b4566 10.1136/bmj.b4566 20028781

[B11] Lofgren S Ljunggren G Brommels M No ticking time bomb: hospital utilisation of 28,528 hip fracture patients in Stockholm during 1998–2007 Scand J Public Health 2010 38 4 418 425 10.1177/1403494810370232 20413586

[B12] Rydholm A Ljunggren G Grafström M Strömberg L Dementia, delirium and other co-morbid conditions in acute hip fracture care – traditions, attitudes and local policies rather than actual state guide diagnose making? Vårdi Norden 2005 25 4 25 2923882294

[B13] Lokk J Borg S Svensson J Persson U Ljunggren G Drug and treatment costs in Parkinson’s disease patients in Sweden ActaNeurol Scand 2012 125 2 142 147 10.1111/j.1600-0404.2011.01517.x 21470194

[B14] Carlsson AC Wandell PE de Faire U Hellenius ML Prevalence of hypertension in immigrants and Swedish-born individuals, a cross-sectional study of 60-year-old men and women in Sweden J Hypertens 2008 26 12 2295 2302 10.1097/HJH.0b013e32831391c3 19008708

[B15] Wandell PE Carlsson AC de Faire U Hellenius ML Prevalence of blood lipid disturbances in Swedish and foreign-born 60-year-old men and women in Stockholm, Sweden NutrMetab Cardiovasc Dis 2011 21 3 173 181 10.1016/j.numecd.2009.09.007 19939652

[B16] Carlsson AC Theobald H Wandell PE Health factors and longevity in men and women: a 26-year follow-up study Eur J Epidemiol 2010 25 8 547 551 10.1007/s10654-010-9472-2 20623324

[B17] Nilsson J Ostling S Waern M Karlsson B Sigstrom R Guo X Skoog I The 1-month prevalence of generalized anxiety disorder according to DSM-IV, DSM-V, and ICD-10 among nondemented 75-year-olds in Gothenburg, Sweden Am J Geriatr Psychiatry 2012 20 11 963 972 10.1097/JGP.0b013e318252e749 22549369

[B18] Wallerblad A Moller J Forsell Y Care-seeking pattern among persons with depression and anxiety: a population-based study in Sweden Int J Family Med 2012 2012 895425 22655197 10.1155/2012/895425 PMC3357962

[B19] Wandell PE Gafvels C Patients with type 2 diabetes aged 35–64 years at four primary health care centres in Stockholm County, Sweden. Prevalence and complications in relation to gender and socio-economic status Diabetes Res Clin Pract 2004 63 3 195 203 10.1016/j.diabres.2003.08.011 14757291

[B20] Gale EA Gillespie KM Diabetes and gender Diabetologia 2001 44 1 3 15 10.1007/s001250051573 11206408

[B21] Jansson SP Andersson DK Svardsudd K Prevalence and incidence rate of diabetes mellitus in a Swedish community during 30 years of follow-up Diabetologia 2007 50 4 703 710 10.1007/s00125-007-0593-4 17268796

[B22] Wandell PE Carlsson A Steiner KH Prevalence of diabetes among immigrants in the Nordic countries Curr Diabetes Rev 2010 6 2 126 133 10.2174/157339910790909404 20201798

[B23] Patten SB Stuart HL Russell ML Maxwell CJ Arboleda-Florez J Epidemiology of major depression in a predominantly rural health region Soc Psychiatry Psychiatr Epidemiol 2003 38 7 360 365 12861441 10.1007/s00127-003-0651-2

[B24] Andersson D Magnusson H Carstensen J Borgquist L Co-morbidity and health care utilisation five years prior to diagnosis for depression. A register-based study in a Swedish population BMC Publ Health 2011 11 552 10.1186/1471-2458-11-552 PMC3144006 21749713

[B25] Egan BM Zhao Y Axon RN US trends in prevalence, awareness, treatment, and control of hypertension, 1988–2008 JAMA 2010 303 20 2043 2050 10.1001/jama.2010.650 20501926

[B26] Kearney PM Whelton M Reynolds K Muntner P Whelton PK He J Global burden of hypertension: analysis of worldwide data Lancet 2005 365 9455 217 223 15652604 10.1016/S0140-6736(05)17741-1

[B27] Joffres MR Hamet P MacLean DR L'Italien GJ Fodor G Distribution of blood pressure and hypertension in Canada and the United States Am J Hypertens 2001 14 1 Pt 1 1099 1105 11724207 10.1016/s0895-7061(01)02211-7

[B28] Mancia G De Backer G Dominiczak A Cifkova R Fagard R Germano G Grassi G Heagerty AM Kjeldsen SE Laurent S 2007 Guidelines for the management of arterial hypertension: The Task Force for the Management of Arterial Hypertension of the European Society of Hypertension (ESH) and of the European Society of Cardiology (ESC) Eur Heart J 2007 28 12 1462 1536 17562668 10.1093/eurheartj/ehm236

[B29] Vasan RS Beiser A Seshadri S Larson MG Kannel WB D'Agostino RB Levy D Residual lifetime risk for developing hypertension in middle-aged women and men: the Framingham heart study JAMA 2002 287 8 1003 1010 10.1001/jama.287.8.1003 11866648

[B30] Marques-Vidal P Tuomilehto J Hypertension awareness, treatment and control in the community: is the ‘rule of halves’ still valid? J Hum Hypertens 1997 11 4 213 220 10.1038/sj.jhh.1000426 9185025

[B31] Wolf-Maier K Cooper RS Banegas JR Giampaoli S Hense HW Joffres M Kastarinen M Poulter N Primatesta P Rodriguez-Artalejo F Hypertension prevalence and blood pressure levels in 6 European countries, Canada, and the United States JAMA 2003 289 18 2363 2369 10.1001/jama.289.18.2363 12746359

[B32] Anandan C Nurmatov U van Schayck OC Sheikh A Is the prevalence of asthma declining? Systematic review of epidemiological studies Allergy 2010 65 2 152 167 10.1111/j.1398-9995.2009.02244.x 19912154

[B33] Osborne ML Vollmer WM Linton KL Buist AS Characteristics of patients with asthma within a large HMO: a comparison by age and gender Am J Respir Crit Care Med 1998 157 1 123 128 10.1164/ajrccm.157.1.9612063 9445289

[B34] Weiner P Magadle R Massarwa F Beckerman M Berar-Yanay N Influence of gender and inspiratory muscle training on the perception of dyspnea in patients with asthma Chest 2002 122 1 197 201 10.1378/chest.122.1.197 12114358

[B35] Ballardini N Kull I Lind T Hallner E Almqvist C Ostblom E Melen E Pershagen G Lilja G Bergstrom A Development and comorbidity of eczema, asthma and rhinitis to age 12: data from the BAMSE birth cohort Allergy 2012 67 4 537 544 10.1111/j.1398-9995.2012.02786.x 22335548

[B36] Wandell PE Carlsson AC Sundquist K Johansson SE Sundquist J Total mortality among levothyroxine-treated women with atrial fibrillation in Swedish primary health care Int J Cardiol 2011 152 1 147 148 10.1016/j.ijcard.2011.07.066 21851994

[B37] Hjerpe P Merlo J Ohlsson H Bengtsson Bostrom K Lindblad U Validity of registration of ICD codes and prescriptions in a research database in Swedish primary care: a cross-sectional study in Skaraborg primary care database BMC Med Inform Decis Mak 2010 10 23 10.1186/1472-6947-10-23 20416069 PMC2864192

[B38] Forrest CB Primary care in the United States: primary care gatekeeping and referrals: effective filter or failed experiment? BMJ 2003 326 7391 692 695 10.1136/bmj.326.7391.692 12663407 PMC152368

[B39] Nazir A Papita R Anbalagan VP Anjana RM Deepa M Mohan V Prevalence of diabetes in Asian Indians based on glycated hemoglobin and fasting and 2-h post-load (75-g) plasma glucose (CURES-120) Diabetes Technol Ther 2012 14 8 665 668 10.1089/dia.2012.0059 22823754

