# Supplementary material for: High prevalence of diagnosis of diabetes, depression, anxiety, hypertension, asthma and COPD in the total population of Stockholm, Sweden – a challenge for public health
Source: BMC Public Health. 2013 Jul 18;13:670. doi: 10.1186/1471-2458-13-670 (PMC3724714; doi:10.1186/1471-2458-13-670)
Supplement: Additional file 1: Figure S1 — Wenn diagrams of reported diagnoses: A) diabetes, C) depression, D) anxiety/phobia, E) hypertension F) asthma, and G) COPD. Primary care = PC, Specialist outpatient care = SOC, Inpatient care = IC. [file 1471-2458-13-670-S1.doc]

**Supplementary Figure 1**
Wenn diagrams of reported diagnoses: A) diabetes, C) depression, D) anxiety/phobia, E) hypertension F) asthma, and G) COPD. Primary care = PC, Specialist outpatient care = SOC, Inpatient care = IC.

A) Per cent of the 72190 diagnosed with diabetes in 2011

PC 56.1 %

SOC 20.2 %

IC 8.8 %

4.7 %

4.7 %

1.9 %

3.6 %

B) Per cent of the 51096 diagnosed with depression in 2011

C) Per cent of the 40096 diagnosed with anxiety/fobia in 2011

PC 56.5 %

SOC 29.8 %

IC 4.1 %

4.5 %

3.2 %

0.8 %

1.1 %

D) Per cent of the 149506 diagnosed with hypertension in 2011

PC 51.6 %

SOC 33.8 %

IC 3.4 %

6.5 %

3.1 %

0.8 %

0.9 %

PC 70.6 %

SOC 5.4 %

IC 10.6 %

3.1 %

1.9 %

1.5 %

7.0 %

E) Per cent of the 46044 diagnosed with asthma in 2011

PC 55.9 %

SOC 28.9 %

IC 5.2 %

6.1 %

1.6 %

0.7 %

1.7 %

1. Per cent of the 19397 diagnosed with COPD in 2011

PC 53.6 %

SOC 10.9 %

IC 15.0 %

4.0 %

4.2 %

4.4 %

8.0 %
